# The ‘Ironclad friendship’ of China-Cambodia, lays the first step in the foundation of early diagnosis and treatment of asymptomatic congenital heart Defects- A multi-national screening and intervention project, 2017–2020

**DOI:** 10.1186/s12872-023-03314-8

**Published:** 2023-06-07

**Authors:** Honglin Song, Xi Li, Jiang Lu, Junjie Song, Teng Wang, Min Gao, Xingyi Zhang, Min Ma, Yi Shi, Jiayu Fang, Hongchen Fu, Huadan Wang, Lin Duo, Mingjing Tang, Linhong Pang

**Affiliations:** 1 Fuwai Yunnan Cardiovascular Hospital, Affiliated Cardiovascular Hospital of Kunming Medical University, Kunming, China; 2 School of Public Health, Kunming Medical University, Yu Hua Street Chun Rong Road, Cheng Gong New City, Kunming, China; 3 National Clinical Research Center for Cardiovascular Diseases, State Key Laboratory of Cardiovascular Disease, Fuwai Hospital, National Center for Cardiovascular Diseases, Chinese Academy of Medical Sciences, Peking Union Medical College, Beijing, China; 4 Central China Subcenter of the National Center for Cardiovascular Diseases, Zhengzhou, China; 5 School of Public Health, Chongqing Medical University, Jinyun Campus, Huxi Town, Shapingba District, Chongqing, China

**Keywords:** Congenital heart disease, Prevalence, China, Cambodia, Asymptomatic, Children

## Abstract

**Background:**

Congenital heart disease (CHD) is the leading cause of mortality in childhood worldwide. However, a large number of children with CHD are not diagnosed promptly in low- and middle-income regions, due to limited healthcare resources and lack the ability of prenatal and postnatal ultrasound examinations. The research on asymptomatic CHD in the community is still blank, resulting in a large number of children with asymptomatic CHD can not be found and treated in time. Through the China-Cambodia collaborative health care initiative, the project team conducted research, screened children’s CHD through a sampling survey in China and Cambodia, collected relevant data, and retrospectively analyzed the data of all eligible patients.

**Objectives:**

The project aimed to evaluate the prevalence of asymptomatic CHD in a sample population of 3-18years old and effects on their growth status and treatment outcomes.

**Methods:**

We examined the prevalence of ‘asymptomatic CHD’ among 3-18years old children and adolescents at the township/county levels in the two participating. A total of eight provinces in China and five provinces in Cambodia were analyzed from 2017 to 2020. During 1 year follow-up after treatment, the differences in heights and weights of the treated and control groups were evaluated.

**Results:**

Among the 3,068,075 participants screened from 2017 to 2020, 3967 patients with asymptomatic CHD requiring treatment were identified [0.130%, 95% confidence interval (CI) 0.126 -0.134%]. The prevalence rate of CHD ranged from 0.02 to 0.88%, and was negatively related to local per capita GDP (p = 0.028). The average height of 3310 treated CHD patients were 2.23% (95% CI: -2.51%~-1.9%) lower than that of the standard group and the average weight was − 6.41% (95% CI: -7.17%~-5.65%) lower, the developmental gap widening with advancing age. One year after treatment, the relative height difference remained comparable while that, in weight was reduced by 5.68% (95% CI: 4.27% ~7.09%).

**Conclusions:**

Asymptomatic CHD now is often overlooked and is an emerging public health challenge. Early detection and treatment are essential to lower the potential burden of heart diseases in children and adolescents.

**Supplementary Information:**

The online version contains supplementary material available at 10.1186/s12872-023-03314-8.

## Background

Congenital heart defects (CHD) are the leading cause of mortality in childhood worldwide [1], affecting 13.3 million people worldwide in 2019 [2]. CHD accounts for more than 40% of deaths related to congenital defects and causes a serious public health burden, especially in low- and middle-income countries, including China and Southeast Asian countries [3–6]. The prevalence of congenital heart disease in China has increased 24-fold from 2015 (0.201‰) to 2019 (4.905‰) [7]. Early detection, followed by surgery or transcatheter intervention,should greatly reduce mortality and morbidity [8]. However, a significant number of asymptomatic patients with CHD are overlooked in antenatal ultrasound and neonatal examinations, resulting in poor prognosis or undernourished and compromised growth status [9].

In many aspects, asymptomatic CHD remains largely understudied. One major issue is the underdiagnosis of the disease, which often leads to serious hypoxia, shock, acidosis, pneumonia, and other complications including death [10–11]. Pulse oximetry is a highly specific and moderately sensitive test for detection of critical CHD with very low false-positive rates [12–13]. The use of pulse oximetry for newborn screening has led to remarkable improvements in detection in recent years but is still not widely available in low and middle-income countries [12–14]. Result from a meta-analysis suggest that a quarter of asymptomatic patients with critical CHDs remain undiagnosed due to its 76% sensitivity of pulse oximetry [14]. Furthermore, there is no data to the knowledge of the authors, or in scientific literature on the potential health effects and treatment outcomes in asymptomatic patients, including–how CHD influences growth status in childhood and strategies to overcome the condition. The above information is crucial in the development of public health policies and resource allocation in the sector of pre-school and early childhood health services.

‘The Children’s CHD Free Screening Project’ in China [15]and the ‘Love Heart Journey Project’[16] in Cambodia, two screening and intervention programs for asymptomatic children and adolescents with CHD, have been implemented since January 2017. The primary goal of this study was to determine the prevalence and subtypes of asymptomatic CHD by screening children in kindergarten, primary, middle and high schools via cardiac auscultation and ultrasound. The second objective was to determine the patient growth status and efficacy of treatment in narrowing the gaps in growth and development.

## Methods

### Design overview and study cohort

In this study, we examined the prevalence of ‘asymptomatic CHD’ among 3–18 years old children and adolescents at the township/county level. The study area incorporated different districts in the two participating nations, China and Cambodia. The author’s affiliated institution (Fuwai Yunnan Cardiovascular Hospital) is located in Yunnan province, which is the largest specialized hospital for cardiovascular disease in southwest China. We selected most counties in Yunnan Province (on our way to cover all areas), as well as relatively remote and poor areas in 7 provinces in western China with local screening needs as screening sites. A total of eight western provinces in China comprising 74 counties and 700 townships were included inthe study. On average, approximately 7.3 million children are residents in each of these selected provinces in China, and the population density in the study areas is around 200 individuals per square kilometer. With the cooperation of an intergovernmental program, a ‘Love Heart Journey Project’ was launched in Cambodia in January 2018. Five provinces of Cambodia with an average of 87 children per square kilometer were selected for the study as the whole process required the commitment and, support of the local governing boards at different administrative/prefectural levels in Cambodia. A map of the study area incorporating the provinces and, counties of both nations is shown (Additional file 1). Far-flung and underdeveloped areas with limited medical resources were included as part of the study plan. Children from kindergarten were thoroughly evaluated at our screening camps. Those diagnosed with CHD and eligible for higher center treatment received appropriate surgical or interventional therapy at a specialist cardiology center.

The overall response rate from the participating was 98.2%. Patients who underwent treatment at tertiary cardiology centers, such as Fuwai Yunnan Cardiovascular Hospital had 95% of their medical costs covered by health insurance schemes offered by governments and philanthropic foundations. The parents of the patients were followed up for at least one year following their discharge from postsurgical intervention. Geographic information system (GIS) was applied to generate a prevalence map of CHD. We further explored the associations among highprevalence clustering patterns of CHD, height and weight growth gap patterns.

### Procedures

The screening team consisted of a cardiologist or cardiac surgeon, three specially trained nurses, two ultrasound specialists, and local community members. For some planned screening sites with a large number of children, the screening team added additional 3–5 cardiologists and nurses to cope with the task. As part of the screening process, a standardized physical examination was initially performed by qualified practice nurses who were vigilant for specific signs (such as abnormal pulse, clubbing fingers, pedal edema, fluid overload, rapid breathing and poor posture) [17]. During this time, detailed history of the child’s mental status, and breathlessness during feeding leading to poor weight gain and easy fatigability were noted [17]. An experienced nurse or a qualified doctor then auscultated the heart in the aortic, pulmonary, tricuspid, and mitral areas with stethoscope B type (Suzhou Yuyue Medical Technology Co., Ltd, China), during which any cardiac souffle or a functional murmur was considered abnormal (Additional file 2). For patients with abnormal heart sounds, further echocardiography was performed using ultrasound Phillip CX 50, Probe S5-1, systematically and comprehensively to assess structural and hemodynamic changes in the heart, from various views, including(a). parasternal long axis, (b). parasternal short axis (at apical, papillary muscle, mitral valve, aortic valve levels), (c). apical (two, three, four, five-chamber), (d). subcostal, and (e). suprasternal views. Various jet flows, pressures, volumes, and chamber areas in both the systole and diastole phases were measured. Based on results obtained by specialists in echocardiography, the cardiologist or cardiac surgeon determined whether the clinical phenotype warranted surgical or interventional treatment, which was ultimately provided at Fuwai Yunnan Cardiovascular Hospital.

### Data collection and measurement

The centers recorded all forms of CHD, mainly, patent ductus arteriosus (PDA Q21.051), tetralogy of Fallot (TOF), atrial septal defect (ASD Q21. 102), and ventricular septal defect (VSD Q21.001, Q21.102), along with other types according to International Classification of Diseases (ICD 10) [18]. With the electronic medical record database of children and adolescents, basic characteristics were extracted, including gender, age, height and weight at admission, CHD subtypes (ASD, VSD, PDA, TOF, and others), after treatment adverse reactions (squatting, shortness of breath after activity, dyspnea, cyanosis, or syncope). We also collected information about annual family income, health insurance, and place of residence through face-to-face interviews with parents.

The parents of treated children and adolescents were contacted one year after discharge to obtain information about their health conditions, including death, reoperation, symptoms, ultrasound manifestations, current weight and height, school attendance, as well as feeding, physical development, and psychological status. To define the natural environment and social development characteristics of patients at each study site, we also searched grey literature, such as statistical yearbooks, for information on average altitude and annual per capita gross domestic product (GDP) in 2017 [19–20].

### Statistical analysis

For categorical variables, frequencies and percentages were calculated while for continuous variables, median [interquartile range (IQR)] values were determined. To estimate the prevalence of asymptomatic CHD and its subtypes in screening, we divided the number of identified cases by the number of screened individuals. The prevalence in the two countries (China and Cambodia), was compared along with subtype fractions among three age groups (3–6 years, 7–12 years, and 13–18 years). The differences between groups were visualized and tested using box plots and the Kruskal-Wallis test. Scatter plots and fitting lines were applied to illustrate the correlation between the prevalence and environmental (altitude) and socioeconomic characteristics (per capita GDP) across counties.To determine the height and weight gaps of patients, we calculated the absolute and relative differences with the age-specific Chinese standard lines [21]. Additionally, the changes in relative gaps during follow-up were calculated by subtracting the baseline from the 1-year value.Patients with missing data were excluded from the analysis. All studies were conducted with SAS 9.4 (SAS Institute Inc., Cary, NC, USA).

## Results

### Prevalence of asymptomatic CHD

A total of 3,068,075 participants were screened (3,015,470 in China and 52,605 in Cambodia), which resulted in the identification of 3967 [0.130%, 95% confidence interval (CI) 0.126 -0.134%] children and adolescents with asymptomatic CHD that needed treatment (Additional file 3).

A total of 3,015,470 children were screened from 7,756 schools in 700 villages in China, among which 3,842 children were diagnosed with CHD. Table 1 shows that the prevalence of asymptomatic CHD was highest in Northwest China (0.201%). The prevalence of asymptomatic CHD was significantly lower in China than Cambodia (0.127%, 95% CI: 0.123 − 0.132% vs. 0.238%, 95%CI: 0.198- 0.283%, p < 0.001). Among the 64 screening sites, the county/district-level prevalence of asymptomatic CHD ranged from 0.022 to 0.883%, which was negatively correlated with local per capita GDP (p = 0.028), but not significantly related to local average altitude (p > 0.05) (Fig. 1).

**Table 1:** Prevalence of CHD in different screening regions

Country	Region	Number of townships	Number of screening schools	Screening children	Confirmed case of CHD	Prevalence rate (%)
**China**	Western Yunnan	97	1332	390,355	504	0.129
	Southwest Yunnan	189	1674	713,905	1071	0.150
	Northwest Yunnan	65	384	130,257	150	0.115
	Central Yunnan	43	629	253,011	232	0.092
	Northeast Yunnan	193	3086	1,251,676	1410	0.113
	Southern Yunnan	27	184	63,091	122	0.193
	Southwest China^#^	56	230	123,006	172	0.140
	Northwest China^#^	30	237	90,169	181	0.201
	**Total**	700	7756	3,015,470	3842	0.127
**Cambodia**	5 provinces in total	NA	80	52,605	125	0.238

Abbreviation: CHD congenital heart disease

NA: Indicates that the data was not available

# The screening centers in 7 provinces other than Yunnan Province were scattered, so they were categorized as “Southern China provinces” and “Northwestern China provinces”

**Fig. 1:**
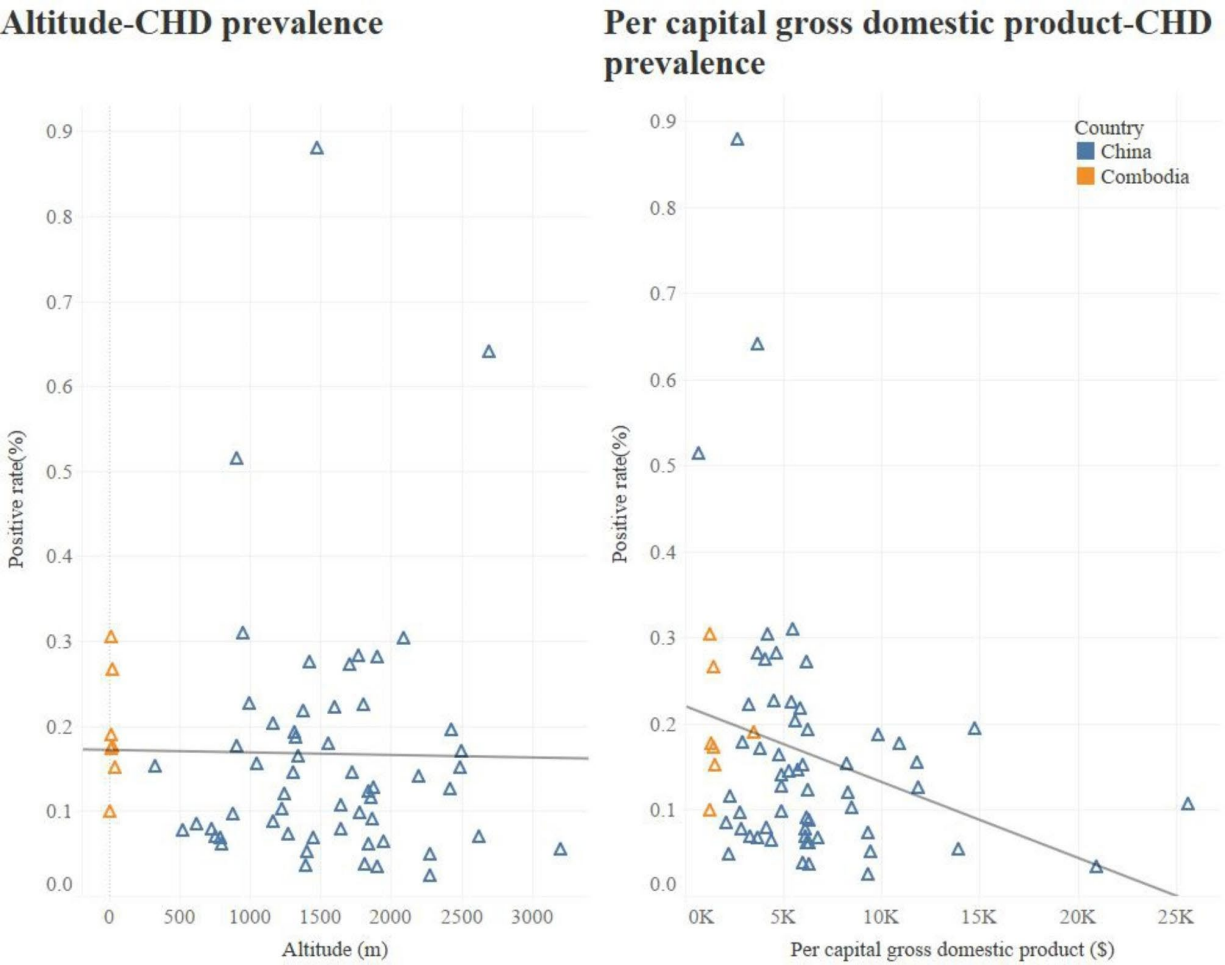
Correlations of CHD prevalence across counties/districts with local per capital GDP and altitude Abbreviations: CHD: indicates congenital heart disease

### Patient characteristics

Among 3310 children with CHD who underwent surgical treatment, 1 child died and 835 were lost to follow-up. Therefore, 2474 children with CHD were followed up (Additional file 3). The median age was 7.0 (3.0 to 11.0) years and 56.4% were female, among which ASD (946, 0.030%, 95% CI: 0.028 − 0.033%), VSD (703, 0.022%, 95% CI: 0.023 -0.024%), and PDA (366, 0.012%, 95% CI: 0.011 − 0.013%) were the most common subtypes (Table 2). Overall, 89.4% of patients residing in rural areas, 31.1% had an annual household income of less than 10 000 Yuan (equivalent to 1587 USD), and 96.7% had social health insurance (Table 2). Compared with patients from Cambodia, those in China were more likely to have an annual household income over 1587 USD, and social health insurance (both p < 0.001).

**Fig. 2:**
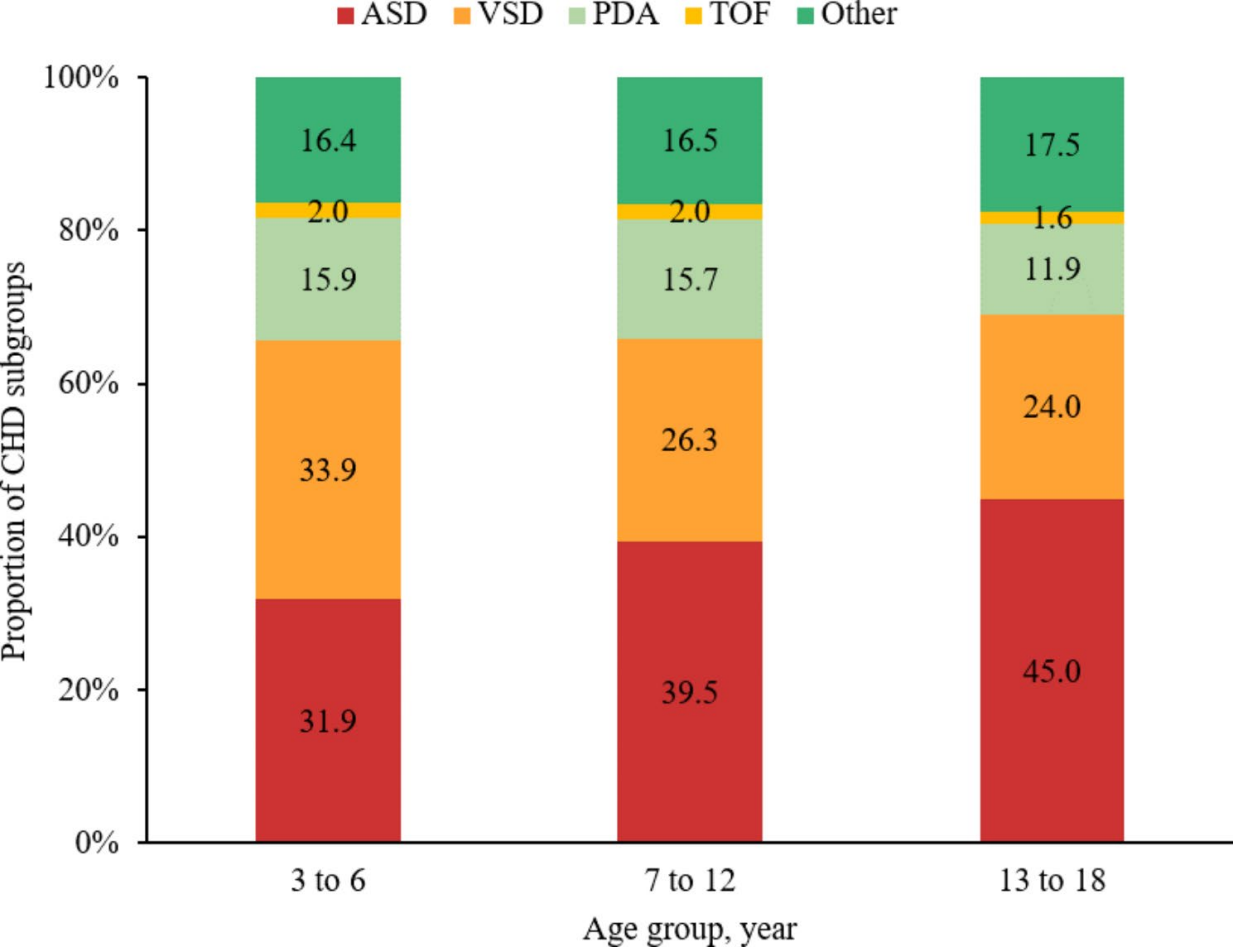
Proportion of CHD subtypes across age groups Abbreviations: CHD: congenital heart disease; PDA: patent ductus arteriosus, TOF: tetralogy of Fallot, ASD: atrial septal defect, VSD: ventricular septal defect

Among the major subtypes of CHD identifiedthe fraction of ASD increased from 31.9% (95% CI: 28.8 − 35.0%) in patients aged 3–6 years to 45.0% (95% CI: 41.4 − 48.9%) in patients aged 13–18 years (p < 0.001), while fraction of VSD [from 33.9% (95% CI: 30.7 − 37.0%) to 24.0% (95% CI: 20.7 − 27.4%)] and PDA [from 15.8% (95% CI: 13.4 − 18.3%) to 11.9% (95% CI: 9.4 − 14.5%)] (both p < 0.05) (Fig. 2).

### Growth gaps

The average height of the patient group requiring treatment was 2.84 (95% CI:2.39 ~ 3.28) cm lower than the standard also presenting as a relative difference of -2.23% (95% CI: -2.54% ~-1.93%). The average patient weight was 1.89 (95% CI:1.59 ~ 2.18) kg lower relative to the standard weight presenting as a relative difference of -6.41% (95% CI: -7.17%~-5.65%). The height (8.88 vs. 2.84 cm, p = 0.0054) and weight gaps (8.59 vs. 1.87 kg, p < 0.001) were more significant in Cambodian than those in Chinese patients.

**Fig. 3:**
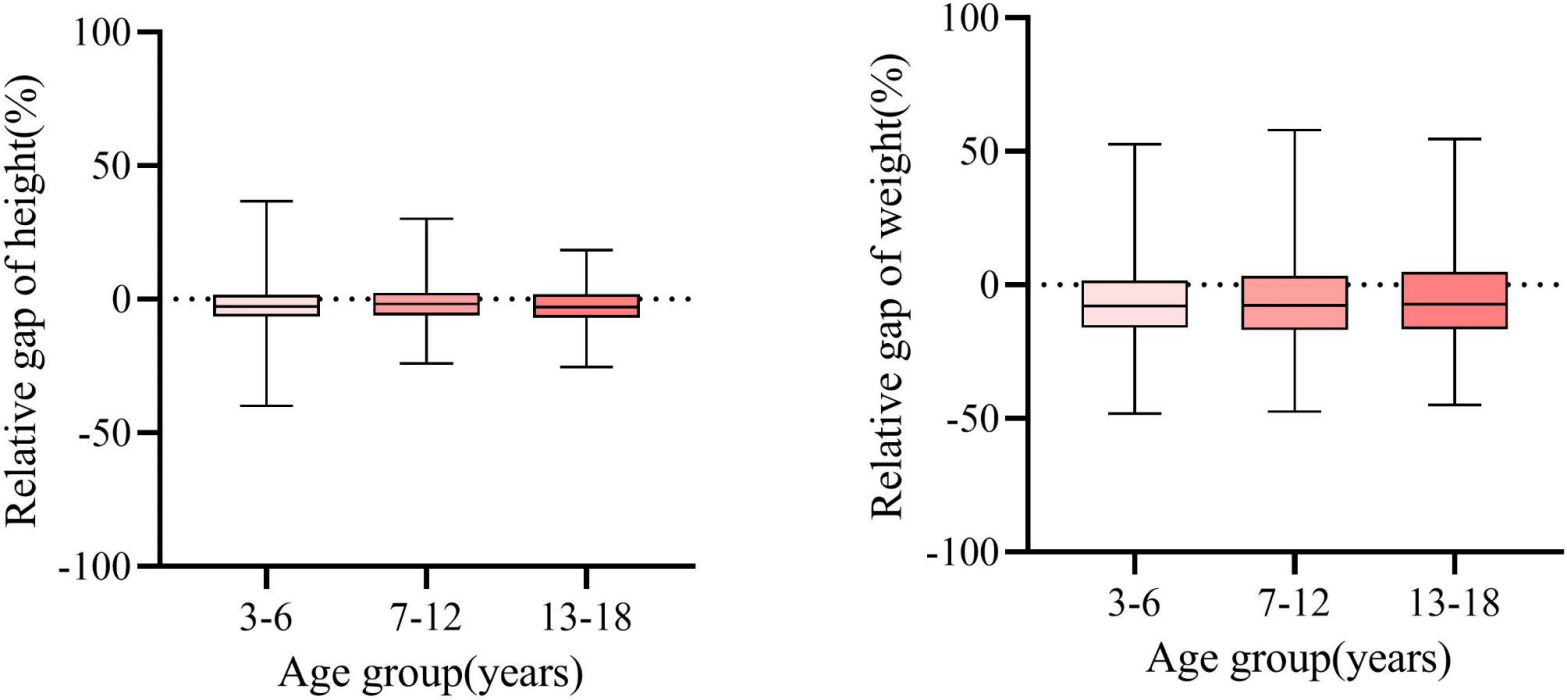
Relative growth gaps across baseline age groups “Relative height or weight gap” = (height or weight of the CHD child divided by the standard height or weight of Chinese child) − 1. Therefore, a value greater than 0 indicated that the child with CHD was developing in accordance with Chinese child growth standards, otherwise the opposite is true

The growth gaps remained across the baseline age groups. The relative gaps in height and weight were − 2.51% (-3.01%~-2.00%) and − 7.24% (-8.41%~-6.08%) for patients aged 3–6 years at diagnosis, -1.91% (-2.38%~-1.44%) and − 5.94% (-7.18%~-4.69%) for those aged 7–12 years, and − 2.59% (-3.06%~-2.13%) and − 5.88% (-7.50%~-4.26%) for 13–18 years old patients (p > 0.05 for all) (Fig. 3).Height and weight gaps were similar, for patients with different subtypes of CHD (Additional file 4).

### Recovery after treatment

After the treatment period, 3 (0.1%) patients died during the 1-year follow-up, while 2474 (74.7%) reported their growth situations. In this cohort, the relative height gap persisted from − 2.23% (95% CI: -2.54%~-1.93%) at baseline to -2.98% (95% CI: -3.46%~-2.50%) 1 year later, with an insignificant change of -0.80% (95% CI: -1.39%~-0.21%); while the relative weight gap was 5.68% lower (95% CI: 4.27%~7.09%) [from − 6.41% (95% CI: -7.17%~-5.65%) to -0.76% (95% CI: -1.88%~0.35%)]. These patterns were consistent between the Chinese and Cambodian patient populations.

**Fig. 4:**
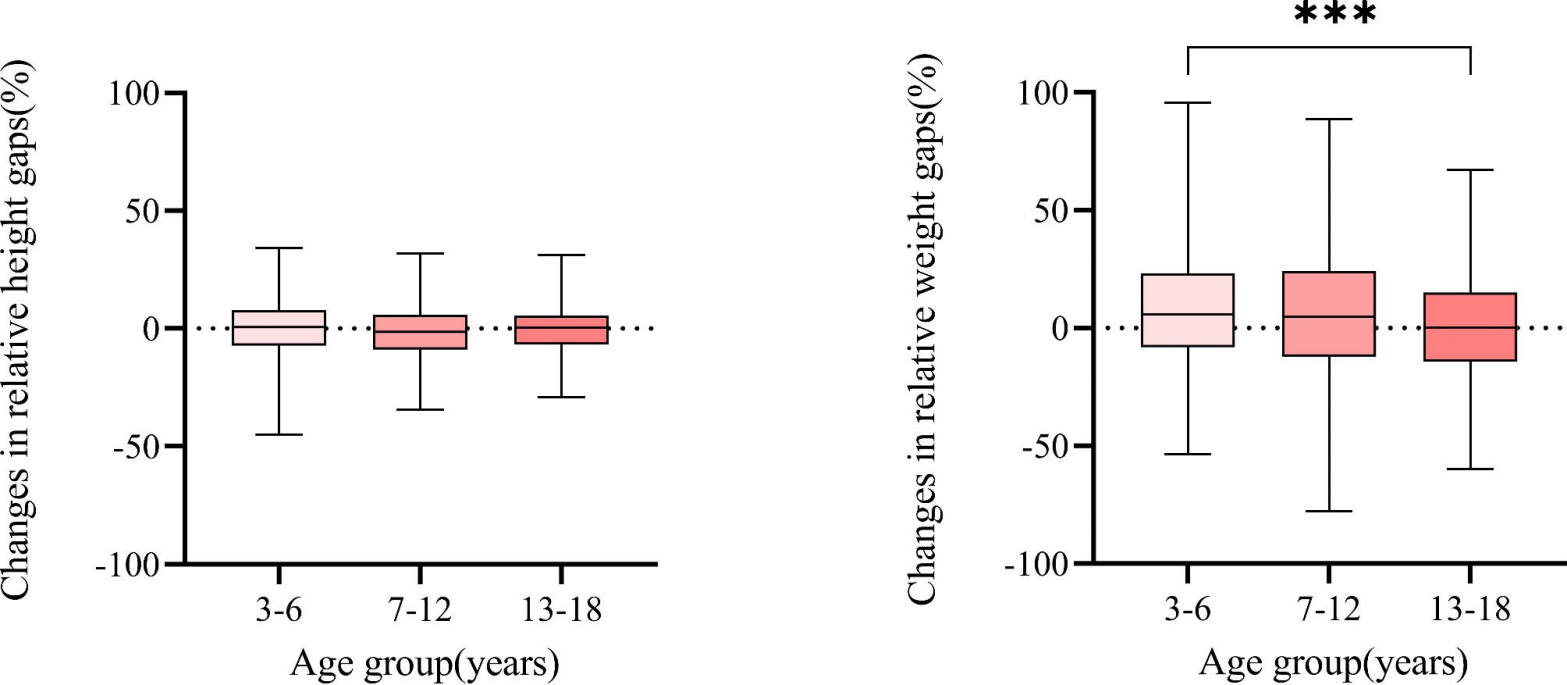
Catching up in growth across baseline age groups “Relative height or weight gap” was both calculated at baseline and at follow-up. “Change in relative height or weight gap” = “Relative height or weight gap at baseline” - “Relative height or weight gap at follow-up”

The “catching-up” steps were larger in patients who were younger during the time of treatment. In patients aged 3–6 years, changes in relative height and weight gaps were 0.09% (95% CI: -0.96%~1.14%) and8.91% (95% CI: 6.70%~11.10%). In contrast, in patients aged 13–18 years, changes in relative height and weight gaps were − 0.81% (95% CI: -1.87%~0.25%) and 0.71% (95% CI: -1.79%~3.21%) (Fig. 4). The changes in growth gaps related to different major subtypes of CHD are presented in Additional file 4.

## Discussion

According to data obtained from screening projects involving over 3 million people in China and Cambodia from January 2017 to Jan. 2020, 0.12% of 3-18-year-old children and adolescents had asymptomatic CHD that required treatment, with rates being higher in less well-developed areas. Patients with this condition experienced delayed growth in height and weight, but early detection and treatment could significantly improve development and outcomes.

In addition to being the largest screening project on CHD, this study contributes to the literature in several aspects. We observed a notable prevalence of asymptomatic CHD in children and adolescents requiring treatment. CHD prevalence was markedly lower relative to that reported in earlier screening studies (0.38 -0.49%)[22–23], but it should be noted that the patients identified in our study are a particularly overlooked group, as the majority of current routine prenatal (e.g. ultrasound) and neonatal (e.g. pulse oximetry) CHD screenings tests focus on critical CHD [24–26]. Moreover, the prevalence of CHD was higher in less developed areas, indicating a higher misdiagnosis rate, which may be attributed to the lack of skilled personnel and advanced facilities for diagnosis and treatment [27–28],as well as parents’ willingness to undergo early CHD screening.Considerable evidence indicates that in the majority of developing countries, achieving the goal of early diagnosis and treatment remains a challenge, which significantly hampers overall population development [28–30]. In developing countries, the lack of sufficient healthcare resources for universal screening generally impedes the development of the entire population [31–32]. This vicious cycle between ‘poverty caused by illness’ and ‘poverty caused by CHD’ needs to beurgently broken [33].

Furthermore, even in cases with no major symptoms, CHD can cause a lag of growth during childhood and adolescence. In addition, children with CHD are at risk of acute and chronic malnutrition due to difficulties in feeding and poor digestion and absorption of nutrients [34–35]. Previous studies on the growth and development of children with CHD revealed smaller effects on weight in school-age children than pre-schoolers, but this finding was attributed to survival bias [36, 37]. Our data suggest that both height and weight gaps related to CHD similarly persist with age across countries. Nevertheless, it is reasonable to expect that the relative gaps widen with patient growth. A survival bias could also affect age-dependent trends in gaps, since the fraction of severe CHD cases among the common subtypes (i.e., VSD and PDA) decreased as patients got older, implying that some of these patients may not be able to continue their education or even die.

Third, early treatment can substantially reduce the growth delay of children and adolescents with asymptomatic CHD. Several studies have shown that after CHD surgery, thecardiac functions of children improve, along with nutritional intake growth and development levels gradually approach those of their healthy counterparts [38–39]. In our study, the weight and height of children with suspected CHD improved after treatment, Highlighting the importance of timely detection and treatment of asymptomatic coronary heart disease. The potential for improving the growth trajectory of the patient is significantly greater with earlier intervention.Treatment effects are expected to influence future physical functions, psychological status, and quality of life in addition to anthropometric parameters [40–41],which calls attention to the need for longer-term observations in this large cohort.

Our study paves the way for a more comprehensive analysis of asymptomatic CHD, especially in middle- and low-income countries. Our findings have significant implications for the development of health policies in these areas. While recognizing the importance of CHD screening, simple and effective technical protocols are necessary for conducting large-scale projects. Cardiovascular auscultation has been confirmed as the most valuable screening method for CHD [42]. This procedure, combined with echocardiography, has allowed us to scale up CHD screening across countries. The key to improving health outcomes in subsequent prompt CHD treatment. However, surgery for CHD remains an advanced technique that is not universally available. Only three hospitals in Cambodia can perform this procedure. Although interventional treatment for CHD is widely adopted in many areas, expensive angiography equipment is required. In Cambodia, only 10 such systems are available. In recent years, new procedures for structural heart disease such as ASD, PDA, and, VSD, which are under echo guidance only, have been established to reduce potential injury related to fluoroscopy and angiography [43–45]. These novel techniques are of particular value for access to treatment in less developed regions, due to their independence from cath laboratories.

Several potential limitations need to be considered when interpreting the findings of the current study. Telephone survey methods may have potential bias leading to skewing of results. Follow-up on CHD from the field is therefore essential. Moreover, the retrospective design of the study raises concerns about the accuracy of recall. However, the concordances between the study and past findings on height and weight growth in children with congenital heart disease give credibility to our findings the effects of CHD on growth and development over the long term require further study and follow-up. Due to field screening time constraints, we failed to collect the complete socio-demographic information on children without CHD besides total numbers, the confirmed CHD cases data available were not sufficient to support the regression models and further adjust for confounding variables.

## Conclusions

To our knowledge, this is the largest community-based congenital heart disease screening project worldwide. This study indicates of many CHD left over in the community, large-scale CHD screening benefits and brought dawn to low and middle-income countries to reduce the potential high burden of CHD. Further development of surgical and interventional treatment capacities in these countries plays a critical role in improving the growth trajectory of disadvantaged patients.

**Table 2:** Characteristics of patients with congenital heart disease, overall and by country

	Overall	China	Cambodia	P value
Demographic and socio-economic				
Female	56.35 (1394/2474)	56.56 (1384/2447)	44.44 (12/27)	0.207
Age, year (Median and IQR)	7.0 (3.0–11.0)	7.0 (3.0–11.0)	8.0 (6.0–13.0)	0.016
Household income: 10 000 RMB (1587 USD) or above per year	68.88 (1704/2474)	69.47 (1700/2447)	14.81 (4/27)	< 0.001
Social medical insurance	96.68 (2392/2474)	97.75 (2392/2447)	0.00 (0/27)	0.163
Rural residence	89.41 (2212/2474)	89.29 (2185/2447)	100.00 (27/27)	0.106
**Clinical profile at admission**				
Subtypes of CHD				< 0.001
Patent ductus arteriosus	14.79 (366/2474)	14.91 (365/2447)	3.70 (1/27)	
Tetralogy of Fallot	1.40 (34/2474)	1.14 (28/2447)	22.22 (6/27)	
Ventricular septal defect	28.41 (703/2474)	28.36 (694/2447)	33.33 (9/27)	
Atrial septal defect	38.24 (946/2474)	38.45 (941/2447)	18.52 (5/27)	
Other	17.19 (425/2474)	17.12 (419/2447)	22.22 (6/27)	
Adverse reactions	34.16 (845/2474)	33.75 (826/2447)	70.37 (19/27)	< 0.001

Values in the table indicate percentages (numerator/denominator) unless otherwise noted.

Abbreviations CHD: congenital heart disease; IQR: inter quartile range.

## Electronic supplementary material

Below is the link to the electronic supplementary material.

Additional File 1: Project sites

Additional File 2: Auscultation technical protocol and auscultation location

Additional File 3: Flow chart of selected participants

Additional File 4: Height and weight gaps before and after treatment

## Data Availability

Original data tied to individuals, locations, and times are considered personally identifiable health information. These data cannot be shared. Aggregated data are available to the extent allowed by a data use agreement. The corresponding authors affirm that the manuscript is an honest, accurate, and transparent account of the study, that no important aspect of the study has been omitted, and that any deviations from the study plan have been explained. Final data for this study can be obtained from the corresponding authors upon reasonable request.

## Abbreviations

PDA: patent ductus arteriosus
TOF: tetralogy of Fallot
ASD: atrial septal defect
VSD: ventricular septal defect
CHD: congenital heart disease
IQR: inter quartile range.
