# Supplementary figures and images for: The ‘Ironclad friendship’ of China-Cambodia, lays the first step in the foundation of early diagnosis and treatment of asymptomatic congenital heart Defects- A multi-national screening and intervention project, 2017–2020
Source: BMC Cardiovasc Disord. 2023 Jun 7;23:288. doi: 10.1186/s12872-023-03314-8 (PMC10246413; doi:10.1186/s12872-023-03314-8)

**Additional file 1. Project sites**

**
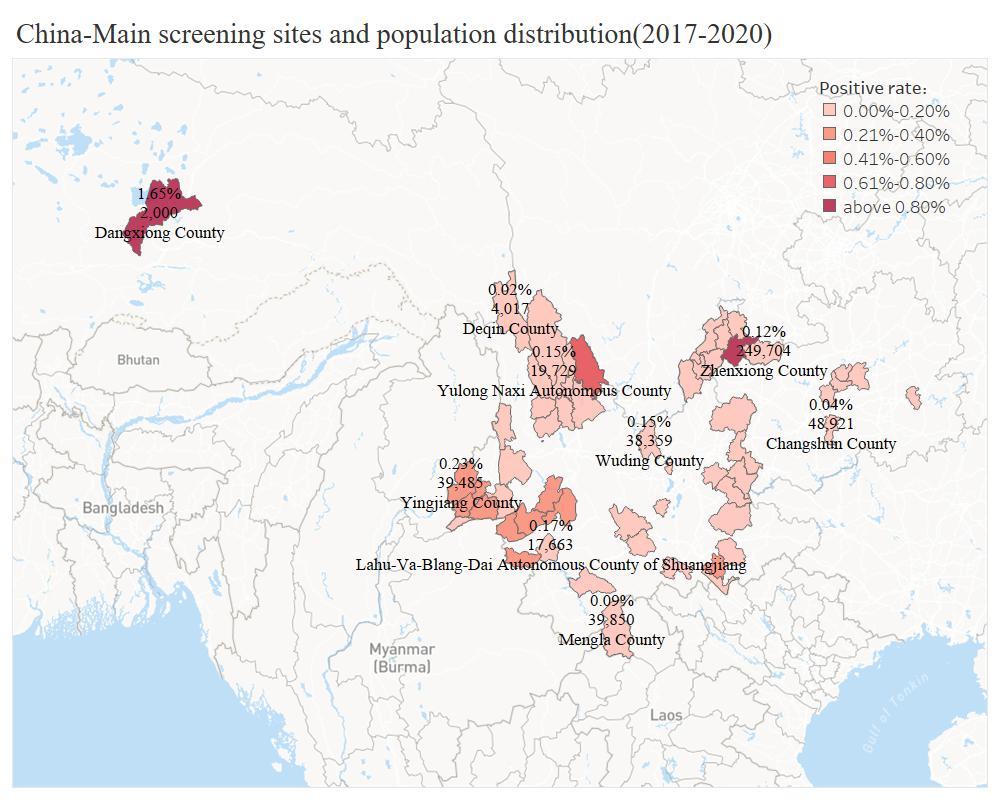
**


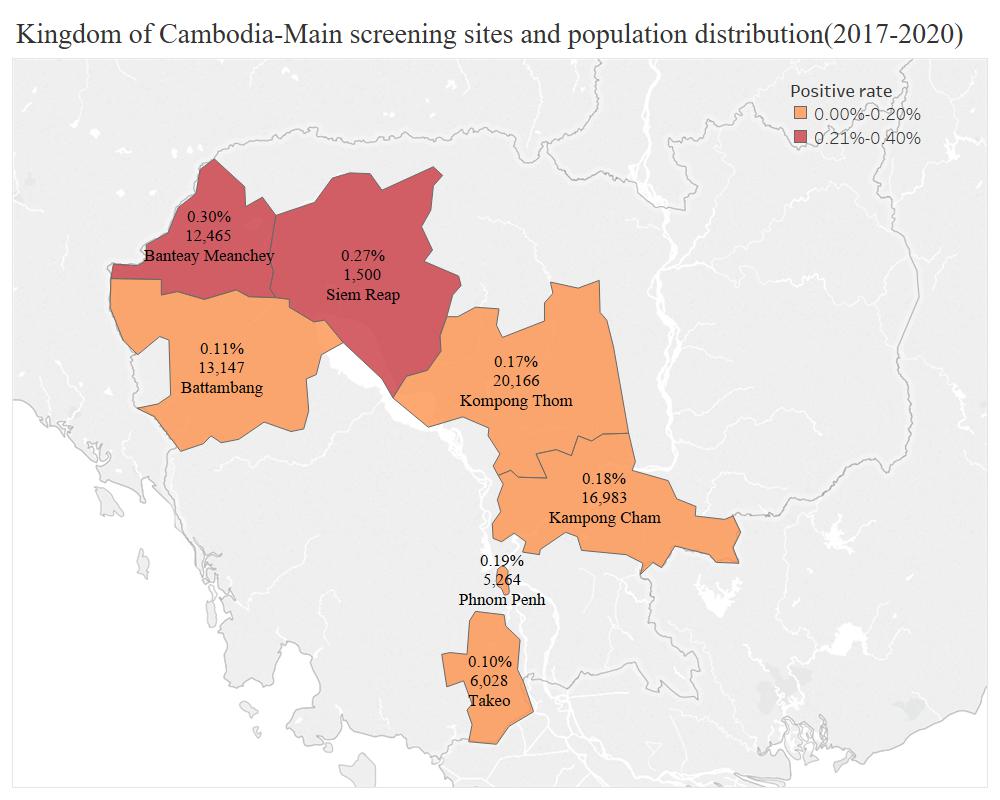

Supplement: Supplementary file 1 — Additional File 1: Project sites [file 12872_2023_3314_MOESM1_ESM.doc]

**Additional file 3. Flow chart of selected participants**


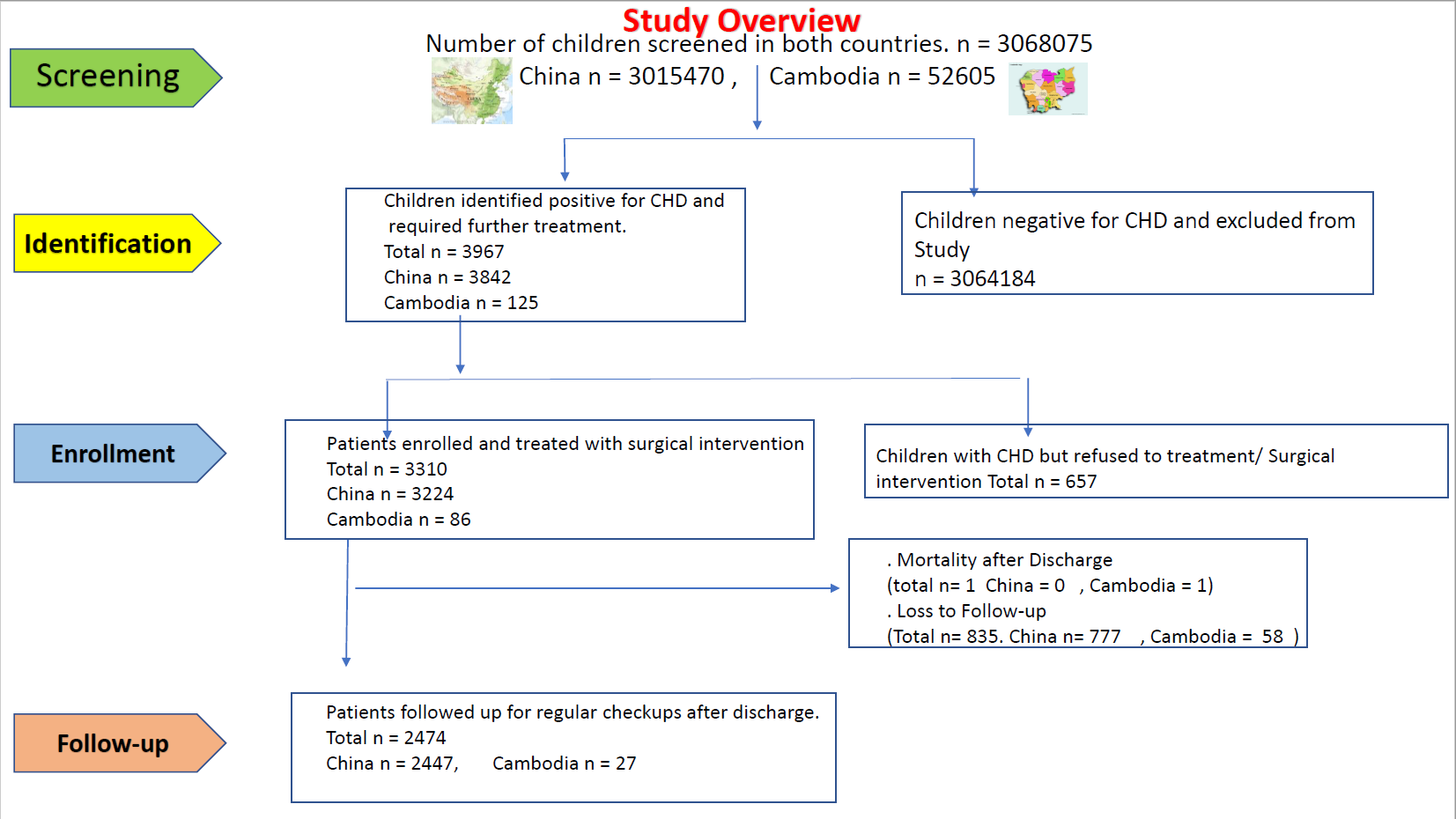

Supplement: Supplementary file 3 — Additional File 3: Flow chart of selected participants [file 12872_2023_3314_MOESM3_ESM.doc]
