# Supplementary material for: The ‘Ironclad friendship’ of China-Cambodia, lays the first step in the foundation of early diagnosis and treatment of asymptomatic congenital heart Defects- A multi-national screening and intervention project, 2017–2020
Source: BMC Cardiovasc Disord. 2023 Jun 7;23:288. doi: 10.1186/s12872-023-03314-8 (PMC10246413; doi:10.1186/s12872-023-03314-8)
Supplement: Supplementary file 2 — Additional File 2: Auscultation technical protocol and auscultation location [file 12872_2023_3314_MOESM2_ESM.doc]

**Additional file 2. Auscultation technical protocol and auscultation location**

Auscultation started at the apex area, pulmonary valve area, aortic valve area, second auscultation area of aortic valve, and tricuspid valve area in sequence to heart rate, rhythm, murmur, extra and friction sound etc., mainly focus on the heart murmur.

**
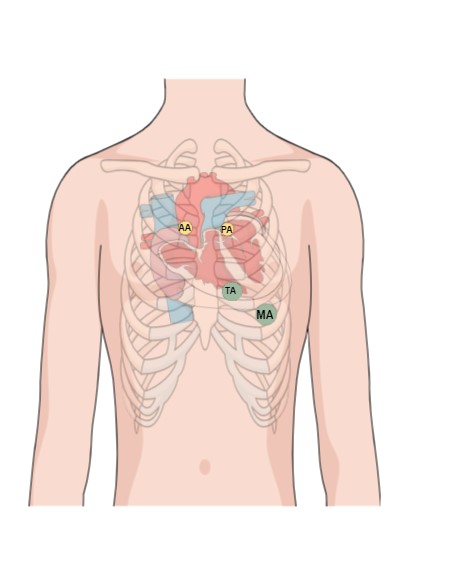
**

**Auscultation areas**- AA: Aortic area, PA: Pulmonary area, TA: Tricuspid area, MA: Mitral area
