# Supplementary material for: The ‘Ironclad friendship’ of China-Cambodia, lays the first step in the foundation of early diagnosis and treatment of asymptomatic congenital heart Defects- A multi-national screening and intervention project, 2017–2020
Source: BMC Cardiovasc Disord. 2023 Jun 7;23:288. doi: 10.1186/s12872-023-03314-8 (PMC10246413; doi:10.1186/s12872-023-03314-8)
Supplement: Supplementary file 4 — Additional File 4: Height and weight gaps before and after treatment [file 12872_2023_3314_MOESM4_ESM.doc]

**Additional file 4**


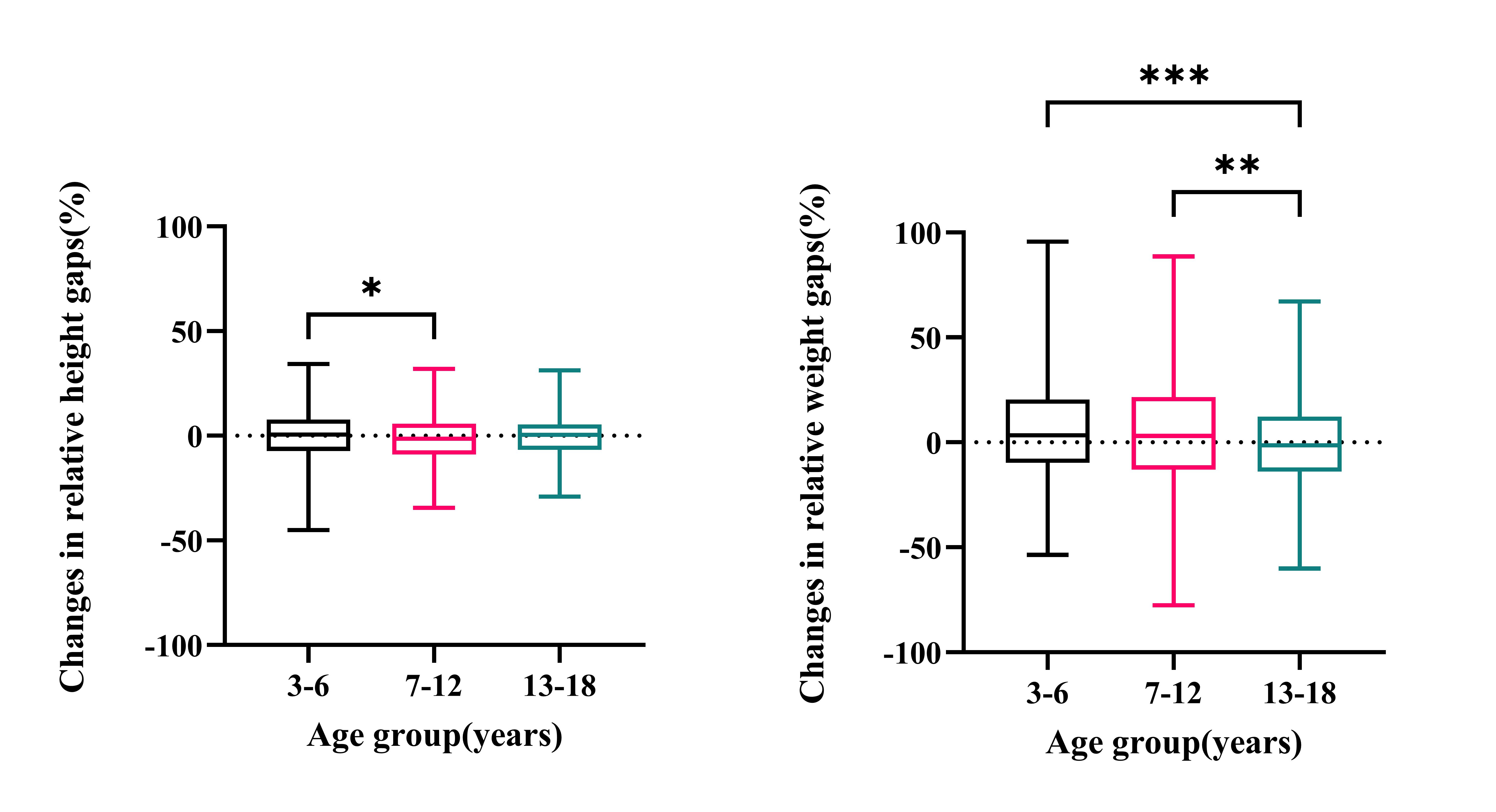


**Figure S1. The growth gaps of patients with different subtypes of CHD**

*Indicates p<0.05, **indicates p<0.01, ***indicates p<0.01


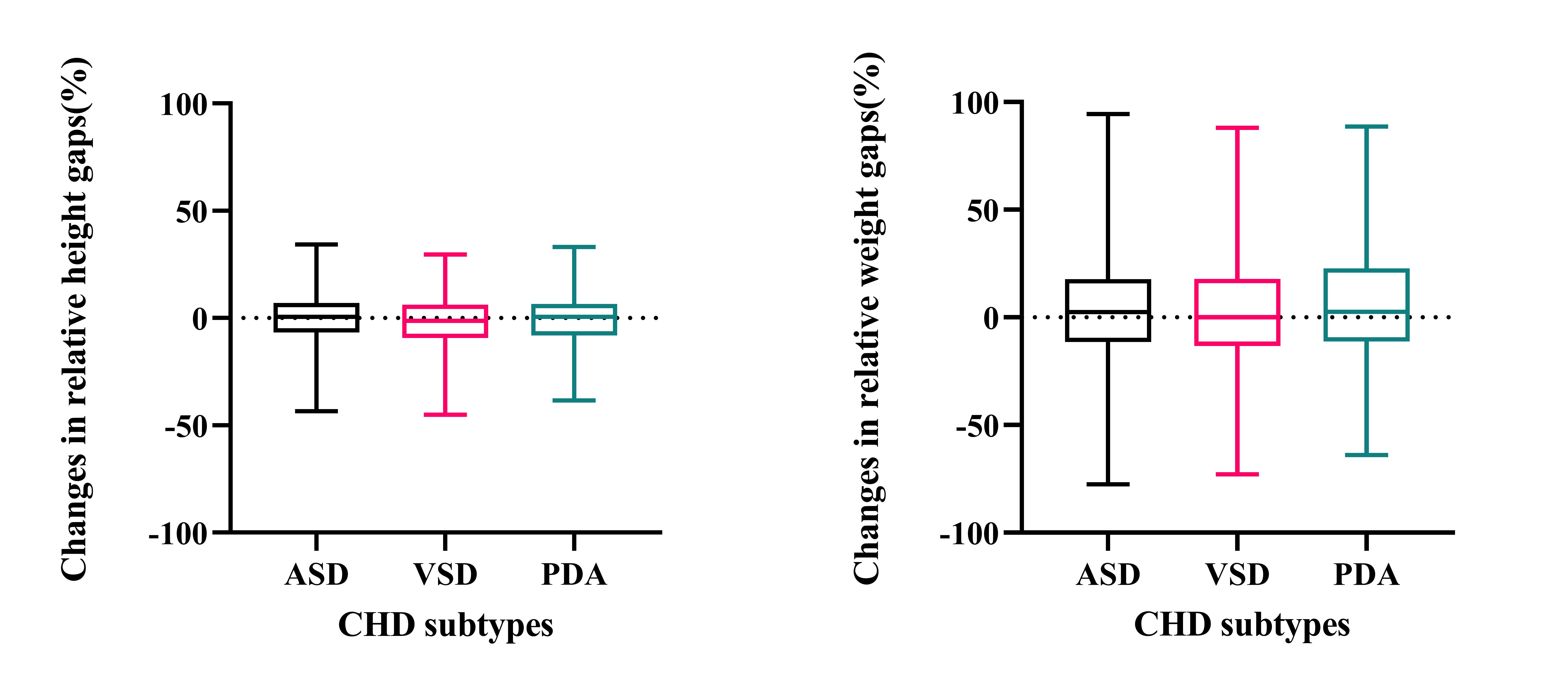


**Figure S2. The changes in relative gaps with different subtypes of CHD**

**Table S1. Height and weight gaps before and after treatment**

|  | | Before treatment | | After treatment | |
| --- | --- | --- | --- | --- | --- |
|  |  | Absolute gaps | Relative gaps | Changes in absolute gaps | Changes in relative gaps |
| Height | ASD | -2.07 (-2.56-1.57) cm | -1.95% (-2.46%~-1.44%) | -0.30 (-1.44~0.85) cm | -0.09% (-1.04%~0.87%) |
|  | VSD | -3.09 (-3.66-2.52) cm | -2.52% (-3.09%~-1.96%) | -1.80 (-3.13~-0.48) cm | -1.28% (-2.37%~-0.19%) |
|  | PDA | -2.46 (-3.22-1.70) cm | -2.11% (-2.80%~-1.43%) | -0.75 (-2.53~1.03) cm | -0.50% (-1.91%~0.91%) |
| Weight | ASD | -1.67 (-2.05-1.30) kg | -6.89% (-8.15%~-5.64%) | 1.209 (0.48-1.94) kg | 5.75% (3.52%~7.97%) |
|  | VSD | -2.14 (-2.53-1.75) kg | -6.57% (-7.97%~-5.18%) | 1.09 (0.26-1.91) kg | 4.60% (2.03%~7.17%) |
|  | PDA | -1.55 (-2.07-1.02) kg | -5.17% (-7.08%~-3.26%) | 1.93 (0.86-3.00) kg | 7.24% (3.73%~10.7%) |
